# Clinicopathological analysis of thyroid carcinomas with the *RET* and *NTRK* fusion genes: characterization for genetic analysis

**DOI:** 10.1007/s00428-024-03777-w

**Published:** 2024-03-12

**Authors:** Yoichiro Okubo, Soji Toda, Mei Kadoya, Shinya Sato, Emi Yoshioka, Chie Hasegawa, Kyoko Ono, Kota Washimi, Tomoyuki Yokose, Yohei Miyagi, Katsuhiko Masudo, Hiroyuki Iwasaki, Hiroyuki Hayashi

**Affiliations:** 1 Department of Pathology, Kanagawa Cancer Center, 2-3-2, Nakao, Asahi-Ku, Yokohama, Kanagawa 241-8515 Japan; 2 Department of Endocrine Surgery, Kanagawa Cancer Center, 2-3-2, Nakao, Asahi-Ku, Yokohama, Kanagawa 241-8515 Japan; 3 Molecular Pathology and Genetics Division, Kanagawa Cancer Center Research Institute, 2-3-2Asahi-Ku, NakaoYokohama, Kanagawa 241-8515 Japan; 4 Department of Pathology, Yokohama Municipal Citizen’s Hospital, 1-1 Mitsuzawanishimachi, Kanagawa-Ku, Yokohama, Kanagawa 221-0855 Japan

**Keywords:** Thyroid carcinoma, *RET*, *NTRK*, *BRAF*, Decalcification, NGS

## Abstract

Thyroid carcinomas exhibit various genetic alterations, including the *RET* and *NTRK* fusion genes that are targets for molecular therapies. Thus, detecting fusion genes is crucial for devising effective treatment plans. This study characterized the pathological findings associated with these genes to identify the specimens suitable for genetic analysis. Thyroid carcinoma cases positive for the fusion genes were analyzed using the Oncomine Dx Target Test. Clinicopathological data were collected and assessed. Among the 74 patients tested, 8 had *RET* and 1 had *NTRK3* fusion gene. Specifically, of the *RET* fusion gene cases, 6 exhibited “*BRAF*-like” atypia and 2 showed “*RAS*-like” atypia, while the single case with an *NTRK*3 fusion gene presented “*RAS*-like” atypia. Apart from one poorly differentiated thyroid carcinoma, most cases involved papillary thyroid carcinomas (PTCs). Primary tumors showed varied structural patterns and exhibited a high proportion of non-papillary structures. Dysmorphic clear cells were frequently observed. *BRAF* V600E immunoreactivity was negative in all cases. Interestingly, some cases exhibited similarities to diffuse sclerosing variant of PTC characteristics. While calcification in lymph node metastases was mild, primary tumors typically required hydrochloric acid-based decalcification for tissue preparation. This study highlights the benefits of combining morphological and immunohistochemical analyses for gene detection and posits that lymph node metastases are more suitable for genetic analysis owing to their mild calcification. Our results emphasize the importance of accurate sample processing in diagnosing and treating thyroid carcinomas.

**Supplementary Information:**

The online version contains supplementary material available at 10.1007/s00428-024-03777-w.

## Introduction

Thyroid carcinomas exhibit various genetic alterations [1], among which the fusion genes are important targets for molecular therapies [2–4]. Determining the presence or absence of these fusion genes is essential for developing novel therapeutic strategies. However, conducting comprehensive genetic analyses in every case is difficult because of technical and economic constraints [5, 6]. In Japan, since 2022, the Oncomine Dx Target Test (for thyroid carcinomas) has been approved for the genetic characterization of advanced or recurrent thyroid carcinomas. This has facilitated widespread genetic analysis of thyroid carcinomas using the Oncomine Dx Target Test, which is expected to be performed at many hospitals. Based on this advancement, we conducted detailed pathological examinations of thyroid carcinomas in which the *RET* and *NTRK3* fusion genes were identified using the Oncomine Dx Target Test. This study aimed to elucidate the specific pathological characteristics associated with the *RET* and *NTRK3* fusion genes and effectively identify cases likely to exhibit one or both of these fusion genes in routine diagnostic work. Additionally, we aimed to characterize the specimens that were most suitable for genetic analysis.

## Materials and methods

In the present study, we enrolled patients with thyroid carcinoma from the Kanagawa Cancer Center (Yokohama, Japan) who were tested using the Oncomine Dx Target Test between May 2022 and October 2023. The rationale for using the Oncomine Dx Target Test, which is approved for use in Japan for patients with advanced or recurrent thyroid carcinoma, was driven by clinical reasons (the test was outsourced). In addition to detecting the *BRAF, RAS*, and other mutations, it also targets fusion genes for identification, including *ABL1*, *ALK*, *AXL*, *BRAF*, *ERBB2*, *ERG*, *ETV1*, *ETV4*, *ETV5*, *FGFR1*, *FGFR2*, *FGFR3*, *MET*, *NTRK1*, *NTRK2*, *NTRK3*, *PDGFRA*, *PPARG*, *RAF1*, *RET*, and *ROS1*. Among these cases, those in which the fusion genes were detected were selected. Basic clinicopathological data, including age; sex; type of fusion gene; histological findings; fusion gene partners; tumor, nodes, and metastases (TNM) classification (UICC 8th edition); and outcome, were obtained from electronic medical records and pathological diagnostic reports. The response to radioactive iodine (RAI) therapy follows the American Thyroid Association Guidelines [7]. Subsequently, the specimens were thoroughly reviewed to validate these data and conduct additional pathological analyses. These analyses included assessing the presence or absence of papillary thyroid carcinoma (PTC) nuclei, counting mitoses per 10 high-power fields, determining the presence or absence of squamous solid nests, and performing immunohistochemical analyses using *BRAF* V600E (Clone: VE1), Ki-67 (Clone: 30–9), and Pan-TRK (Clone: EPR17341). PTC nuclei were assessed based on a previous report using a 3-point scoring system, with a score of 2 or higher being defined as PTC nuclei [8]. Furthermore, based on a previous report, we also assessed whether “*BRAF*-like” atypia or “*RAS*-like” atypia was present [9]. The primary tumor was analyzed; however, in cases where the primary tumor could not be obtained, recurrent lymph node metastases or metastases in other organs submitted for the Oncomine Dx Target Test were used. Given the known high frequency of *RET* fusion genes in diffuse sclerosing variant of PTC (DSVPTC) [10–12], we adopted the diagnostic criteria based on a previous report for identifying relevant factors in this study [9]. At the structural level, the patterns of papillary, follicular, and solid/trabecular/insular (STI) structures were analyzed, and their percentages out of the total were evaluated. Only the percentages of papillary, follicular, and STI structures were included, excluding other structures from the denominator. The degree of fibrosis and calcification was also evaluated. The degree of fibrosis was expressed as a percentage of the total tumor lesions. For calcification, two distinct types were evaluated: psammoma bodies (a small, concentrically layered calcification formed around a necrotic tumor cell nucleus, typically found within the tumor stroma and lymphatic vessels [13]), and coarse calcification. Henceforth, the term “calcification” will refer exclusively to the latter, that is, coarse type. Calcification was evaluated on a scale of 0–3B. Each level was defined as follows: 0, no obvious calcification; 1, psammoma bodies or calcified nests that did not require a decalcification procedure; 2A, requiring only ethylenediaminetetraacetic acid (EDTA)-based decalcification for less than half of the specimens; 2B, requiring only EDTA-based decalcification for more than half of the specimens; 3A, requiring hydrochloric acid-based decalcification for less than half of the specimens; 3B, requiring hydrochloric acid-based decalcification for more than half of the specimens. When both EDTA and hydrochloric acid decalcification were present, hydrochloric acid decalcification was prioritized.

## Results

**Fig. 1:**
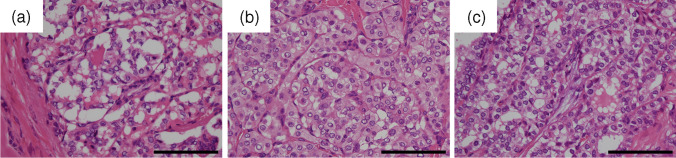
Representative microscopic images of a thyroid carcinoma with fusion genes in the high-power fields. **a** Carcinoma cells in the primary tumor exhibit the characteristic “dysmorphic clear cell.” It was defined as a cell with highly compressed nuclei and clear to pale cytoplasm (Case 2, H&E stain, original magnification × 400, scale bar, 100 µm). **b** Carcinoma cells in lymph node metastases exhibiting typical eosinophilic (pink) or pale-to-clear cytoplasm. The former contributed to the typical pink intranuclear cytoplasmic inclusions, whereas the latter contributed to the washed-out-like intranuclear cytoplasmic inclusions. So-called “*BRAF*-like” atypia was present. Some cells also showed highly compressed nuclei and dysmorphic clear cells. These morphologic characteristics were observed in both decalcified primary tumors and non-decalcified lymph node metastases, indicating morphologic consistency between the different specimens (Case 3, H&E stain, original magnification × 400, scale bar, 100 µm). **c** Carcinoma cells in primary tumors exhibit so-called “RAS-like” atypia characterized by round nuclei, powdery chromatin, and lack of intranuclear cytoplasmic inclusions (Case 7, H&E stain, original magnification × 400, scale bar, 100 µm)

**Fig. 2:**
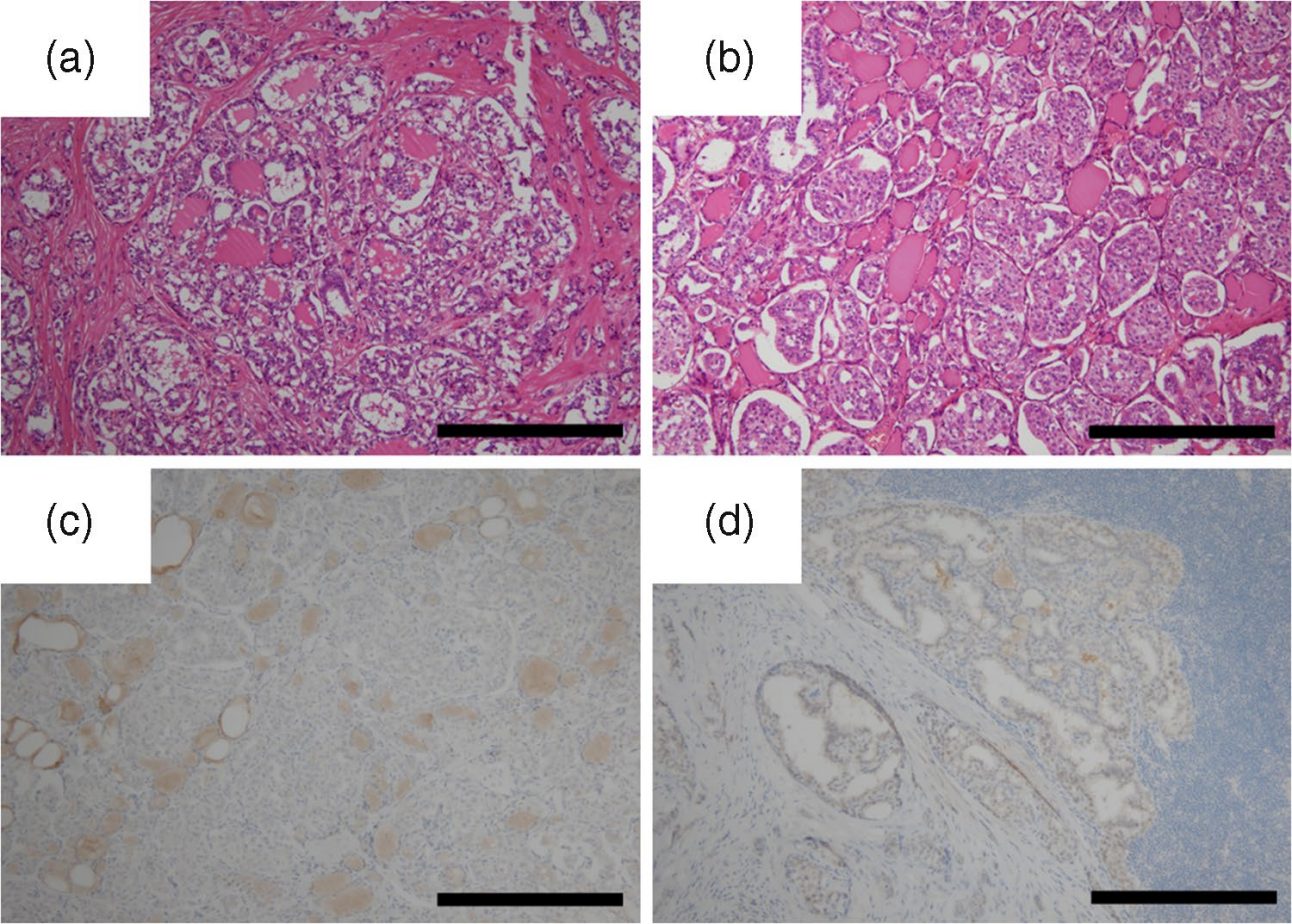
Representative microscopic images of thyroid carcinoma with fusion genes in the middle-power fields. **a** Despite the occurrence of papillary thyroid carcinoma, the cancer cells exhibited follicular structure in addition to papillary structure (Case 6, H&E stain, original magnification × 100, scale bar, 400 µm). **b** Carcinoma cells exhibit small solid or insular structures without the characteristics of poorly differentiated thyroid carcinoma, such as convoluted nuclei, significant mitotic activity, and tumor necrosis (Case 6, H&E stain, original magnification × 100, scale bar, 400 µm). **c** Immunohistochemical staining for *BRAF* V600E. Carcinoma cells showed negative immunoreactivity (Case 6, immunohistochemistry, clone: VE1, original magnification × 100, scale bar, 400 µm). **d** Immunohistochemical staining for Pan-TRK. Carcinoma cells showed negative immunoreactivity (Case 7, immunohistochemistry, clone: EPR17341, original magnification × 100, scale bar, 400 µm)

**Fig. 3:**
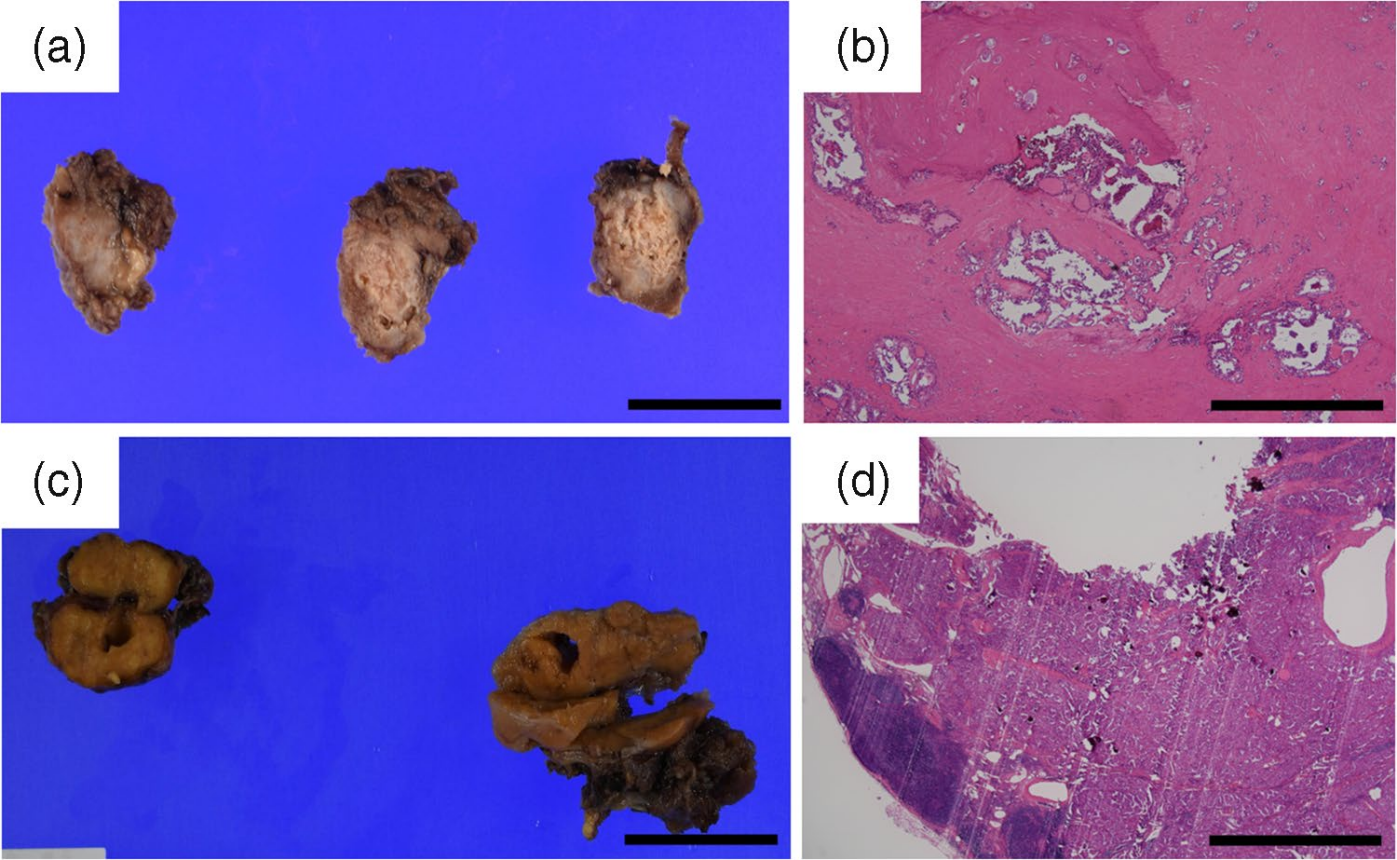
Macroscopic and microscopic images of primary tumors and lymph node metastases in thyroid carcinomas with fusion genes. **a** Macroscopic image of the thyroid gland in Case 7 showing extensive calcification and sclerosis (gross view; scale bar, 30,000 µm). **b** Microscopic image of the thyroid gland in Case 7, showing severe calcification. Owing to the severe calcification, hydrochloric acid-based decalcification was required to prepare the slide (H&E stain, original magnification × 40, scale bar, 1000 µm). **c** Macroscopic image of the lymph node metastasis in Case 3. As the clinician had informed pathologists that there was a large lymph node metastasis, an intentional partial incision was made for proper formalin fixation (gross view; scale bar, 30,000 µm). **d** Microscopic image of the lymph node metastasis in Case 3. The slide has a rough appearance due to the presence of psammoma bodies; however, a decalcification procedure was not required for slide preparation (H&E stain, original magnification × 20, scale bar, 2000 µm)

In our hospital, 74 thyroid carcinoma cases were tested using the Oncomine Dx Target Test between July 2022 and October 2023. This excludes one case in which the test was canceled owing to a low tumor cell count and prior hydrochloric acid decalcification. The histological types are as follows: PTC in 66 cases, follicular thyroid carcinoma (FTC) in three cases, poorly differentiated thyroid carcinoma (PDTC) in four cases, and medullary thyroid carcinoma (MTC) in one case. No anaplastic thyroid carcinomas were present. Genetic analysis revealed that PTC primarily exhibited the *BRAF* V600E mutation (47 out of 66 PTC cases, 71.2%), whereas FTC exclusively showed *RAS* mutations (three out of three FTC cases, 100%). The *RET* fusion gene was detected in eight cases (8/74, 10.8%), whereas the *NTRK3* fusion gene was detected in one case (1/74, 1.4%). No other fusion genes, including *ALK*, *ROS1*, and *PPARG*, were detected. Detailed data are summarized in Supporting Information 1. Among the nine cases exhibiting the fusion genes, the median patient age was 53 years, and the male-to-female ratio was 2:7. None of the nine patients in this study had a history of radiation exposure. For the response to RAI therapy, six cases exhibited a “structural incomplete response,” two cases exhibited an “indeterminate response,” and one case did not receive RAI therapy. In the cases with the fusion genes, primary tumors were confirmed in only seven cases (1, 2, 4, 6, 7, 8, and 9) in our hospital. Moreover, in two cases (3 and 5), we could not obtain the primary tumor because they involved surgeries performed at external hospitals. Most of the patients were diagnosed with PTC. Only one patient (case 9) exhibited lymph node metastases characteristics of a PDTC. All of the obtained cases, except for PDTC in case 9, had PTC nuclei. In addition, detailed examination revealed “*BRAF*-like” atypia in cases 2–4, 6, 8, and 9, and “*RAS*-like” atypia in cases 1, 5, and 7 (Fig. 1b–c). Note that case 9 was assessed only on the primary tumor, as the lymph node metastases had progressed to PDTC. The mitoses of the primary tumors were all < 1 per 10 high-power fields. Lymphatic invasion was observed in two cases (cases 1 and 4; 28.6%, 2/7), whereas vascular invasion was observed in all cases. Squamous solid nests were confirmed in three cases (cases 1, 2, and 4; 42.9%, 3/7). Immunohistochemical examination revealed a median Ki-67 labeling index of 1.3% for primary tumors. All cases were negative for *BRAF* V600E, both in immunoreactivity (Fig. 2c) and mutations. Immunoreactivity of Pan-TRK was negative in case 7 (Fig. 2d), which had the *ETV*6::*NTRK*3 fusion gene. These clinicopathological data are summarized in Table 1. Pathologically, in primary tumors, the median percentage of papillary structures was 50%, and follicular and STI structures were observed (Fig. 2a, b) while lymph node metastases exhibited a more pronounced papillary structure. The prevalence of STI structures in the lymph node groups was low. Moreover, dysmorphic clear cells, identified by highly compressed nuclei and pale-to-clear cytoplasm, were observed to varying degrees in all cases except case 4 (Fig. 1a, b). The median percentage of fibrosis in the primary tumors was 40%. In the lymph node groups, the median percentages were 20% in the N1a group and less than 10% in the N1b group. A decalcification procedure was necessary in the majority case of primary tumors but it was not needed for the N1b lymph node except for one case (Fig. 3a–d and Table 2). Furthermore, in our study, while none of the nine cases completely matched the essential criteria for DSVPTC [9], several distinct characteristics were observed. These included a predominantly young and female patient population and significant occurrence of RAI resistance. Additionally, features such as intraglandular dissemination, lymphatic invasion, squamous solid nests, and/or chronic thyroiditis were confirmed in some cases. Varying degrees of fibrosis and patterns of follicular or STI structures were also observed across many cases. These data are summarized in Supporting Information 2.

**Table 1:** Clinicopathological findings of thyroid carcinomas with the *RET* or *NTRK* fusion genes

	Age	Sex	Histology	Fusion gene partners	TNM classification	Lymphatic invasion	Vascular invasion	Mitosis	Squamoid solid nests	Ki-67 LI	Nuclear findings	*BRAF* V600E (IHC)	Response to radioactive iodine therapy	Outcome
Case 1	16	Female	PTC	*CCDC6*::*RET*	pT1a, pN1b, cM1 (lung)	+	+	< 1	+	6.0	“*RAS*-like” atypia	-	Structural incomplete response	AWD with a follow-up of 11 months
Case 2	22	Female	PTC	*NCOA4*:: *RET*	pT2, pN1b, cM1 (lung)	-	+	< 1	+	1.1	“*BRAF*-like” atypia	-	Structural incomplete response	AWD with a follow-up of 6 months
Case 3	29	Male	PTC	*NCOA4*:: *RET*	pT4a, pN1b, cM1 (lung)	NS					“*BRAF*-like” atypia	-	Indeterminate response	AWD with a follow-up of 3 months
Case 4	51	Female	PTC	*CCDC6*::*RET*	Initial: pT3a, N1b, pM0 Recurrence: rM1 (lung)	+	+	< 1	+	2.9	“*BRAF*-like” atypia	-	Structural incomplete response	AWD with a follow-up of 7 months
Case 5	53	Female	PTC	*ERC1*::* RET*	Initial: unknown Recurrence: rM1 (multiple organs)	NS					“*RAS*-like” atypia	-	ND	DOD with a follow-up of 8 months
Case 6	57	Female	PTC	*CCDC6*::*RET*	pT2, pN1b, cM1 (lung)	-	+	< 1	-	2.8	“*BRAF*-like” atypia	-	Structural incomplete response	AWD with a follow-up of 8 months
Case 7	59	Male	PTC	*ETV6*::*NTRK3*	pT3b, pN1b, cM1 (lung)	-	+	< 1	-	0.0	“*RAS*-like” atypia	-	Indeterminate response	AWD with a follow-up of 9 months
Case 8	70	Female	PTC	*CCDC6*::*RET*	pT3a, pN1a, pM1 (lung)	-	+	< 1	-	0.3	“*BRAF*-like” atypia	-	Structural incomplete response	AWD with a follow-up of 5 months
Case 9	72	Female	PDTC	*ERC1*::* RET*	pT1b, pN1b, rN1 (supraclavicular lymph node), cM0	-	+	< 1	-	1.3	“*BRAF*-like” atypia	-	Structural incomplete response	AWD with a follow-up of 12 months

*PTC*, papillary thyroid carcinoma; *PDTC*, poorly differentiated thyroid carcinoma; + , positive/present; -, negative/absent; < 1, less than 1 per 10 high-power fields; *AWD*, alive with disease; *DOD*, died of disease;* NS*, no specimen. This table summarizes the clinicopathological data of nine thyroid carcinoma harboring the RET or NTRK fusion genes. TNM classifications (UICC 8th edition) are primarily based on pathology (“p”), with “cM1” indicating clinical evidence of distant metastasis. The designation “*r*” denotes recurrences. Pathological assessments primarily reflect the findings of initial surgeries for primary tumors. The number of mitoses per 10 high-power fields is described. “NS” was used for cases lacking primary tumors (cases 3 and 5). However, confirmation of nuclear findings of “*BRAF*-like” atypia or *“RAS*-like” atypia and for *BRAF* V600E immunohistochemical evaluation, metastatic tissues were used in cases 3 and 5 because of the inability to confirm the primary tumor. Case 9, in which only lymph node metastasis met the criteria for poorly differentiated thyroid carcinoma, is noted in the table, with a mitotic count of less than one in the primary tumor. The response to radioactive iodine therapy follows the American Thyroid Association Guidelines, and outcomes are reported in months since the submission to the Oncomine Dx Target Test

**Table 2:** Assessment of structural and morphological fibrosis, and calcification patterns between primary and nodal lesions in thyroid carcinomas

		Structural distribution (%)			Dysmorphic clear cells (10 HPFs)	Fibrosis (%)	Calcification intensity
		Papillary	Follicular	STI			
Case 1	Primary	90	10	0	6	40	2A
	N1a	90	10	0	10	20	1
	N1b	90	10	0	10	20	1
Case 2	Primary	50	10	40	9	20	1
	N1a	90	10	0	0	< 10	1
	N1b	90	10	0	2	< 10	1
Case 3	Primary	NS					
	N1a	NS					
	N1b	60	0	40	8	< 10	1
Case 4	Primary	90	10	0	0	20	3B
	N1a	100	0	0	0	< 10	3B
	N1b	100	0	0	0	< 10	1
Case 5	Primary	NS					
	N1a	NS					
	N1b	NS					
Case 6	Primary	50	20	30	10	40	3A
	N1a	100	0	0	3	< 10	0
	N1b	100	0	0	0	< 10	1
Case 7	Primary	60	40	0	7	80	3B
	N1a	100	0	0	1	30	2B
	N1b	100	0	0	0	10	1
Case 8	Primary	10	90	0	9	40	3B
	N1a	10	90	0	3	60	3B
	N1b	NM					
Case 9	Primary	10	90	0	10	90	3B
	N1a	90	0	10	3	40	3B
	N1b	40	0	60	8	10	3A

*STI*, solid, trabecular, and insular; *HPF*, high-power field; *NM*, no metastasis; *NS*, no specimen. This table provides a detailed account of the structural distribution, dysmorphic clear cell counts in 10 HPFs, fibrosis percentages, and calcification intensity for the primary tumor and N1a and N1b lymph node groups. For Case 3, only the N1b lymph node group data were available because the primary and N1a group surgeries were performed at an external hospital. For Case 5, histological data were not available as both the primary and lymph node surgeries were performed at an external hospital. Case 8 lacked data for the N1b lymph node group owing to the absence of metastasis in that group. The structural patterns were assessed for papillary, follicular, and STI structures, and the proportion of each category out of the total number of observed structures was calculated. Dysmorphic clear cells were reported as the total count observed across 10 HPFs, irrespective of individual field counts. Fibrosis was quantified as a percentage of the entire tumor mass, with instances of mild fibrosis (< 10%) denoted by “ < 10.” Calcification was assessed using a six-point scale: 0, no obvious calcification; 1, psammoma body or calcified nests that did not require decalcification; 2A, requiring only EDTA-based decalcification for less than half of the specimens; 2B, requiring only EDTA-based decalcification for more than half of the specimens; 3A, requiring hydrochloric acid-based decalcification for less than half of the specimens; 3B, requiring hydrochloric acid-based decalcification for more than half of the specimens

## Discussion

Recently, the importance of molecular-targeted drugs in the treatment of thyroid carcinoma has been increasingly recognized [3, 4, 14–16]. Although the significance of *BRAF* inhibitors is well established, the role of targeted therapies for the fusion genes (such as *RET*, *NTRK*, *ALK* fusion genes) is also important [17, 18]. Notably, *BRAF* mutations are mutually exclusive with these fusion genes [19, 20]. Therefore, the efficient detection of these fusion genes is important for the selection of appropriate molecular-targeted therapies. Of course, recent advancements in preoperative molecular testing for genes have significantly aided early thyroid carcinoma diagnosis [1, 3, 15, 16]. However, limitations such as sampling variability and the heterogeneous nature of cancer can affect the comprehensiveness of these tests [21, 22]. The cost of testing is also an issue [21, 23, 24]. Considering economic and insurance constraints, it is important to establish a process that can detect cases with these fusion genes from routine diagnostic work. Our study, which analyzed surgical specimens, complements this approach by providing a more comprehensive molecular and histological assessment.

In thyroid carcinoma, the frequency of *RET* fusion genes ranges from approximately 6 to 10% [25–28], which aligns with our results. Similarly, the frequency of *NTRK* fusion genes ranges from 2 to 7% [26, 29–32]. However, the frequency of *NTRK* fusion genes in our study was lower. This discrepancy could be attributed to several factors. First, in our analysis, we used the Oncomine Dx Target Test, approved in Japan only for advanced or recurrent cases. Second, our study had a comparatively small sample size. Final, the detection of the *NTRK* fusion gene using the Oncomine Dx Target Test could be limited owing to difficulties in covering relevant intron regions, in which the *NTRK* fusion breakpoints commonly occur [33]. The frequency of *ALK* fusion genes in thyroid carcinoma ranges from approximately 1 to 4% [34–36], with a slightly higher frequency of 6% in pediatric PTC cases [37]. However, in our study, we did not detect any *ALK* fusion genes, which may have the same issues as those found with *NTRK* fusion genes. Moreover, our patient cohort included advanced cases, most of which were RAI therapy-resistant. This might introduce a selection bias. In addition, among the 66 PTC cases (10/66, 15.2%), ten exhibited no identifiable genetic alterations. A recent report indicates that the categorization of such PTC cases with no initial driver event identification as “dark matter” PTCs might be premature [9]. The absence of detectable genetic alterations in these cases may be because of limitations of the testing methods and may require a more comprehensive diagnostic approach, if necessary.

In our histological analysis, we observed the presence of non-papillary (follicular and/or STI) structures, compressed nuclei, and cells with clear to pale cytoplasm (reminiscent of dysmorphic clear cells), along with notable fibrosis and calcification. These features were prevalent in several cases, suggesting distinct histological patterns. Given that *BRAF* V600E mutations in papillary thyroid carcinomas are associated with distinct morphological features, including a well-developed papillary architecture [38], it can be hypothesized that non-papillary structures may be more pronounced in thyroid carcinomas with *RET* and/or *NTRK3* fusion genes. In addition, case 7, the sole case with a detected *NTRK*3 fusion gene, exhibited “*RAS*-like” atypia. Each case with a *RET* fusion gene presented distinct morphologic features, with some cases showing “*BRAF*-like” atypia (cases 2–4, 6, 8, and 9) and others exhibiting “*RAS*-like” atypia (cases 1 and 5). This variability in cases with *RET* fusion genes and “*RAS*-like” atypia in cases with *NTRK* fusion genes is consistent with that in a previous report [9] and underscores the morphological distinctions associated with these specific genetic changes. In addition, dysmorphic clear cells were observed in both decalcified primary tumors and non-decalcified lymph node metastases, indicating morphological consistency between the different specimen preparations. Despite the small number of cases, the overall morphology of the cases with the *RET* fusion gene and the *NTRK3* fusion gene was similar. This finding is consistent with that of a previous report on sarcoma cases with these fusion genes [39]. In this study, all cases with detected fusion genes exhibited negative immunoreactivity for *BRAF* V600E. These findings suggested a high probability of the presence of the *RET* or *NTRK* fusion genes in cases with various structural patterns, dysmorphic clear cells, and background fibrosis and/or calcification. However, as confirmed in case 4, the absence of these morphological characteristics does not indicate the absence of these fusion genes, emphasizing the limitations of morphological assessment alone. Nevertheless, the negative immunoreactivity for *BRAF* V600E in all cases indicates that the combination of morphological analysis with immunohistochemistry may improve the prediction for thyroid carcinoma harboring the *RET* and/or *NTRK* fusion genes. It should be noted, however, that Pan-TRK immunohistochemistry may not always yield positive results in cases with the *ETV*6::*NTRK*3 fusion gene, as previously reported [9].

Meanwhile, our cases exhibited some similarities to those of DSVPTC. These included a relatively young patient population and the occurrence of RAI resistance. Intraglandular dissemination, lymphatic invasion, squamous solid nests, and/or chronic thyroiditis were noted in some cases. Additionally, varying degrees of fibrosis and patterns of follicular or STI architecture were observed across many cases. However, it is important to recognize that in Japan, the Oncomine Dx Target Test is used primarily in advanced or recurrent cases. This specific limitation for the use of the test may explain the lack of definite DSVPTC in our series. Despite this, the clinicopathological similarities we observed suggest a potential association between thyroid carcinomas with detected fusion genes and DSVPTC. Furthermore, some reports have suggested that thyroid carcinomas with the *RET* and/or *NTRK* fusion genes tend to be aggressive [28, 29, 40, 41]; our findings indicate a more complex scenario. Although all our cases showed low mitotic activity and a low Ki-67 labeling index, distant metastases were observed in cases 1–8, and a PDTC component was present in case 9. This implies that the level of aggressiveness was not fully captured by these markers.

We would like to shed some light on the challenges and considerations in decalcification processes, particularly in thyroid carcinoma harboring fusion genes and varying degrees of calcification. In our hospital, the choice between EDTA-based and hydrochloric acid-based decalcification depended on the degree of calcification. EDTA-based decalcification is preferred to preserve nucleic acid quality [42, 43], which is important for detecting fusion genes. However, in cases of more severe calcification, which prevents sectioning for pathological diagnosis, hydrochloric acid-based decalcification is required despite the risk of affecting nucleic acid quality [42, 43]. In this study, significant calcification was observed in many primary tumors that required hydrochloric acid-based decalcification. In contrast, calcification in the lymph node metastases, particularly in the N1b group, tended to be mild. These findings suggest that lymph node metastases, particularly those in the N1b group, may be more suitable for genetic analysis of thyroid carcinoma cases. The reason for the mild calcification in the lymph node metastases is unclear. In our study, younger patients, such as those in cases 1 and 2, exhibited mild calcification of the primary tumor. This finding implies that mild calcification in lymph node metastases may reflect their recent occurrence compared to primary tumors. As these metastatic cells are derived from a late-stage primary tumor, their stay in the lymph nodes is relatively short, which may result in mild calcification. However, as lymph nodes are located in adipose tissues and are covered by a capsule [44], it may be necessary to remove the surrounding adipose tissue and make special incisions to obtain the specimen to ensure proper formalin fixation for genetic analysis.

In conclusion, our study has identified unique pathological characteristics associated with the *RET* and *NTRK*3 fusion genes in thyroid carcinoma cases, such as non-papillary structures, compressed nuclei, dysmorphic clear cells, calcification, and similarities with DSVPTC. These characteristics could serve as indicators for considering fusion gene testing. Additionally, our analysis of the varying degrees of calcification between primary tumors and lymph node metastases, particularly in the N1b group, underscores the importance of precise sample processing for efficient genetic analysis. Put together, these insights contribute to a more targeted approach in the molecular diagnosis and treatment of thyroid carcinomas (details in Supporting Information 3).

## Limitations

This study has several limitations. This study involved a limited number of cases and subjective pathology analysis. As the Oncomine Dx Target Test is approved only for advanced or recurrent thyroid carcinoma cases in Japan, there may have been bias in the analyzed cases. Moreover, decisions regarding the selection of decalcification procedures (EDTA- or hydrochloric acid-based) depended on the judgment of the clinical laboratory technicians.

## Supplementary Information

Below is the link to the electronic supplementary material.

Supplementary file1 (DOCX 20 KB)

## Data Availability

The datasets used and/or analyzed during this study are available from the corresponding author upon reasonable request.
