# Supplementary material for: Clinicopathological analysis of thyroid carcinomas with the RET and NTRK fusion genes: characterization for genetic analysis
Source: Virchows Arch. 2024 Mar 12;485(3):509–18. doi: 10.1007/s00428-024-03777-w (PMC11415398; doi:10.1007/s00428-024-03777-w)
Supplement: Supplementary file 1 — Supplementary file1 (DOCX 20 KB) [file 428_2024_3777_MOESM1_ESM.docx]

Supporting Information 1

|  | Papillary thyroid carcinoma (n=66) | Follicular thyroid carcinoma (n=3) | Poorly differentiated thyroid carcinoma (n=4) | Medullary thyroid carcinoma (n=1) |
| --- | --- | --- | --- | --- |
| *BRAF* V600E | 47 (71.2%) | 0 (0%) | 1 (25%) | 0 |
| *RAS* mutation | 1 (1.5%) | 3 (100%) | 1 (25%) | 0 |
| *RET* fusion | 7 (10.6%) | 0 (0%) | 1 (25%) | 0 |
| *RET* mutation | 0 (0%) | 0 (0%) | 0 (0%) | 1 (100%) |
| *NTRK* fusion | 1 (1.5%) | 0 (0%) | 0 (0%) | 0 |
| *ALK* fusion | 0 (0%) | 0 (0%) | 0 (0%) | 0 |
| Genetic alterations were not detected | 10 (15.2%) | 0 (0%) | 1 (25%) | 0 |

b: This Supporting Information 1 provides the genetic analysis results of 74 patients submitted to the Oncomine Dx Target Test. Papillary thyroid carcinoma (PTC) predominantly exhibited the BRAF V600E mutation, while follicular thyroid carcinoma showed RAS mutations alone. Among the analyzed cases, RET and NTRK fusion genes were identified in 9 patients: 8 with PTC and 1 with poorly differentiated thyroid carcinoma. No other fusion genes, including *ALK*, *ROS1*, and *PPARG*, were detected in this cohort (Notably, *PPARG* was not included in the Oncomine Dx Target Test panel). Additionally, there were instances of analysis failures, with 2 cases in DNA-based analysis and 7 in RNA-based analysis.

Supporting Information 2 Assessing agreement with the criteria for diffuse sclerosing variant papillary thyroid carcinoma^a^

|  | Age | Sex | Diffuse involvement of  1 or both lobes of thyroid | Lymphatic invasion in stroma away from main tumor mass | Psammoma bodies in stroma away from main tumor mass | Absence of high-grade features | Numerous  psammoma bodies | Fibrosis (%) | Squamoid  solid nests | Follicular or solid/trabecular/insular architecture (%) | Chronic thyroiditis |
| --- | --- | --- | --- | --- | --- | --- | --- | --- | --- | --- | --- |
| Case1 | 16 | Female | Intraglandular dissemination present | Present | Present | Consistent | Present | 40 | Present | 10 | None |
| Case2 | 22 | Female | Intraglandular dissemination present | None | None | Consistent | None | 20 | Present | 50 | None |
| Case3 | 29 | Male | ND | | | | | | | | |
| Case4 | 51 | Female | Intraglandular dissemination present | Present | Present | Consistent | Present | 20 | Present | 10 | None |
| Case5 | 53 | Female | ND | | | | | | | | |
| Case6 | 57 | Female | None | None | None | Consistent | None | 40 | None | 50 | None |
| Case7 | 59 | Male | None | None | None | Consistent | None | 80 | None | 40 | Present |
| Case8 | 70 | Female | None | None | None | Consistent | None | 40 | None | 90 | Present |
| Case9 | 72 | Female | Intraglandular dissemination present | None | None | Inconsistent | None | 90 | None | 90 | Present |

a: This Supporting Information 2 provides a comprehensive table detailing various characteristics in accordance with the diagnostic criteria for diffuse sclerosing variant papillary thyroid carcinoma by Chou A *et al* (reference number 34*)*. Namely, it provides data such as age, sex, and key characteristics including diffuse involvement of the thyroid lobes, lymphatic involvement, presence of psammoma bodies, absence of high-grade features, percentage of fibrosis, presence of squamoid solid nests, and percentage of follicular or solid/trabecular/insular architecture. Although none of the nine cases fully met the essential criteria for diffuse sclerosing variant of papillary thyroid carcinoma, partial agreement with these criteria was observed and summarized.

Supporting Information 3

Title: Efficient analysis process for papillary thyroid carcinoma with fusion genes

Legend: Review of clinical data, including patient age and disease progression, is essential to identify cases potentially benefiting from genetic analysis. For accurate genetic testing, strategic incisions for tissue samples and removing adipose tissue around lymph nodes may be required. The decision to decalcification procedure should balance nucleic acid preservation against sample visibility. Histologically, features often associated with fusion genes, such as non-papillary structures, dysmorphic clear cells, and psammoma bodies/calcifications, warrant careful examination. Resemblance to the diffuse sclerosing variant of papillary thyroid carcinoma is an important consideration. Assess the nuclear characteristics for signs of “*BRAF*-like” or “*RAS*-like” atypia. The evaluation of *BRAF* V600E mutation immunoreactivity is crucial, as *BRAF* mutations typically exclude the presence of *RET* and/or *NTRK* fusion genes. This immunohistochemical analysis assists in confirming the *BRAF* V600E mutation status. Upon receiving the final genetic analysis results, cases with specific mutations will be re-evaluated. At this stage, a detailed examination of the association between any detected genetic alterations and histological findings is undertaken. This analysis aims to gain detailed insights into the relationship between genetic mutations and histological findings.
